# Early-onset epileptic encephalopathy caused by a reduced sensitivity of Kv7.2 potassium channels to phosphatidylinositol 4,5-bisphosphate

**DOI:** 10.1038/srep38167

**Published:** 2016-12-01

**Authors:** Maria Virginia Soldovieri, Paolo Ambrosino, Ilaria Mosca, Michela De Maria, Edoardo Moretto, Francesco Miceli, Alessandro Alaimo, Nunzio Iraci, Laura Manocchio, Alessandro Medoro, Maria Passafaro, Maurizio Taglialatela

**Affiliations:** 1 Department of Medicine and Health Sciences, University of Molise, Campobasso, Italy; 2 CNR Institute of Neuroscience, Department of Medical Biotechnology and Translational Medicine (BIOMETRA), University of Milan, Milan, Italy; 3 Department of Neuroscience, University of Naples Federico II, Naples, Italy; 4 Centre for Integrative Biology (CIBIO), University of Trento, Trento, Italy; 5 Instituto Biofisika (UPV/EHU, CSIC), Leioa, Spain; 6 Department of Pharmacy, University of Salerno, Fisciano, Salerno, Italy

## Abstract

Kv7.2 and Kv7.3 subunits underlie the M-current, a neuronal K^+^ current characterized by an absolute functional requirement for phosphatidylinositol 4,5-bisphosphate (PIP_2_). Kv7.2 gene mutations cause early-onset neonatal seizures with heterogeneous clinical outcomes, ranging from self-limiting benign familial neonatal seizures to severe early-onset epileptic encephalopathy (Kv7.2-EE). In this study, the biochemical and functional consequences prompted by a recurrent variant (R325G) found independently in four individuals with severe forms of neonatal-onset EE have been investigated. Upon heterologous expression, homomeric Kv7.2 R325G channels were non-functional, despite biotin-capture in Western blots revealed normal plasma membrane subunit expression. Mutant subunits exerted dominant-negative effects when incorporated into heteromeric channels with Kv7.2 and/or Kv7.3 subunits. Increasing cellular PIP_2_ levels by co-expression of type 1γ PI(4)P5-kinase (PIP5K) partially recovered homomeric Kv7.2 R325G channel function. Currents carried by heteromeric channels incorporating Kv7.2 R325G subunits were more readily inhibited than wild-type channels upon activation of a voltage-sensitive phosphatase (VSP), and recovered more slowly upon VSP switch-off. These results reveal for the first time that a mutation-induced decrease in current sensitivity to PIP_2_ is the primary molecular defect responsible for Kv7.2-EE in individuals carrying the R325G variant, further expanding the range of pathogenetic mechanisms exploitable for personalized treatment of Kv7.2-related epilepsies.

Phosphatidylinositol 4,5-bisphosphate (PIP_2_), a negatively charged lipid only found in the inner leaflet of the plasma membrane, regulates several classes of ion channels, with few exhibiting an absolute functional dependence on PIP_2_ levels. Among these, Kv7 voltage-dependent potassium (K^+^) channels only conduct current when membrane PIP_2_ levels achieve critical values^1^,^2^,^3^,^4^. Heteromeric assembly of Kv7.2 and Kv7.3 subunits (encoded by the KCNQ2 and KCNQ3 genes, respectively) underlie the M-current (I_KM_), a slowly activating and deactivating neuronal K^+^ current which regulates excitability in the sub-threshold range for action potential generation, thus contributing to network oscillation and synchronization^5^. Depletion of membrane PIP_2_ upon activation of G_q_-coupled receptors inhibits I_KM_, increasing neuronal excitability^2^,^3^. In addition, PIP_2_ exposure reverses homomeric Kv7.2 and heteromeric Kv7.2 + Kv7.3 current rundown occurring spontaneously in excised patches^3^. Single channel recordings from Kv7 channels of various subunit compositions revealed that when PIP_2_ is depleted the open probability approaches zero; increasing PIP_2_ levels induces a concentration-dependent increase in channel open probability, without changing the single channel conductance, the ionic selectivity, or the number of channels at the plasma membrane^6^.

Mutations in Kv7.2 are responsible for neonatal-onset epileptic diseases with a heterogeneous phenotypic presentation^7^. On the benign end of the spectrum is familial neonatal seizures (BFNS), an autosomal-dominant epilepsy characterized by recurrent seizures beginning in the first days of life and remitting after a few weeks or months, with mostly normal interictal EEG, neuroimaging, and psychomotor development. By contrast, de novo missense Kv7.2 mutations can lead to a severe epileptic encephalopathy (Kv7.2-EE), in which neonates develop pharmacoresistant seizures with distinct EEG and neuroradiological features, and various degrees of developmental delay^8^. De novo missense Kv7.2 mutations are among the most common causes of early-onset EEs^9^,^10^.

Both loss-of-function^11^,^12^ and gain-of-function^13^,^14^ molecular mechanisms have been identified in Kv7.2-EE; understanding the molecular pathogenesis in Kv7.2-EE is crucial to deduce genotype-phenotype correlations which may improve diagnostic, prognostic and therapeutic approaches. In this work, we have explored the molecular pathogenesis of a Kv7.2 mutation (R325G) found recurrently in three cases of Kv7.2-EE with early-onset seizures, burst-suppression pattern at the EEG, and profound global developmental delay^15^,^16^; the same variant has been more recently reported in a fourth patient with atypical presentation (neonatal-onset seizures) of the Kleefstra syndrome, a genetic disorder characterized by intellectual disability, limited or absent speech, hypotonia, synophrys, hypertelorism, and microcephaly^17^. The results obtained suggest that the R325G mutation severely impaired Kv7.2 channel function by reducing channel apparent affinity for PIP_2_; therefore, strategies increasing cellular PIP_2_ levels might provide therapeutic benefit in Kv7.2-EE patients carrying this and, possibly, other mutations affecting PIP_2_-dependent regulation.

## Results

### Functional and biochemical characterization of homomeric and heteromeric channels carrying Kv7.2 R325G subunits

**Figure 1:**
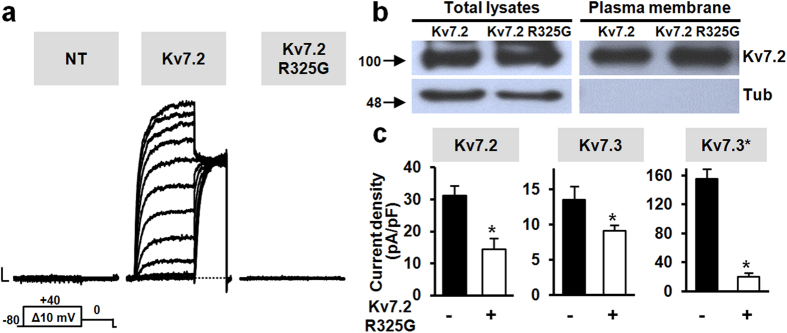
Functional and biochemical characterization of Kv7.2 R325G subunits. (**a**) Macroscopic currents from CHO cells in response to the voltage protocol shown; NT: non-transfected cells. Current scale: 200 pA; time scale: 200 ms. (**b**) Western blot analysis of proteins from total lysates (left) or biotinylated plasma membrane fractions (right) from CHO cells transfected with the indicated constructs. Higher and lower blots were probed with anti-Kv7.2 or anti-α-tubulin antibodies, as indicated. Numbers on the left correspond to the molecular masses of the protein markers. For clarity, the images shown are cropped from full-length gels; these are shown as Supplementary Information (Supplementary Fig. 2). (**c**) Current densities (0 mV) in cells co-expressing Kv7.2 R325G subunits and Kv7.2 (n = 11 and 20 in the absence and in the presence of Kv7.2 R325G subunits, respectively), Kv7.3 (n = 15 and 13 in the absence and in the presence of Kv7.2 R325G subunits, respectively), or Kv7.3* (n = 18 and 17 in the absence and in the presence of Kv7.2 R325G subunits, respectively) subunits. Asterisks indicate values significantly different (p < 0.05) versus respective controls.

Homomeric Kv7.2 channels expressed in Chinese Hamster Ovary (CHO) cells by transient transfection carried robust outward K^+^ currents activating at about −40 mV and showing slow activation and deactivation kinetics, and lack of inactivation. Instead, no current could be recorded from cells transfected with Kv7.2 R325G cDNA; macroscopic current densities at 0 mV in Kv7.2 R325G-transfected and non-transfected cells were identical, being respectively 0.7 ± 0.1 pA/pF and 1.1 ± 0.1 pA/pF (p > 0.05) (Fig. 1a). Despite such dramatic loss of function, Western-blot experiments revealed a similar amount of Kv7.2 or Kv7.2 R325G subunits in both total lysates and plasma membrane-isolated fractions from CHO cells (Fig. 1b); in fact, the average values for the OD_Q2Tot_/OD_Tub_ ratios (in total lysates) and the OD_Q2Biot_/OD_Q2Tot_ ratios (in biotinylated plasma membrane-enriched fractions) were 0.85 ± 0.13 and 1.15 ± 0.11, or 0.71 ± 0.03 and 0.85 ± 0.17, in Kv7.2- or Kv7.2 R325G-transfected cells, respectively (n = 4; p > 0.05).

Having detected a significant plasma membrane expression of non-functional Kv7.2 R325G mutant subunits, their possible inhibitory effects on the currents carried by Kv7.2 and/or Kv7.3 channels were evaluated. As shown in Fig. 1c, current densities at 0 mV in cells co-transfected with Kv7.2 and Kv7.2 R325G cDNAs (1.8 μg + 1.8 μg) was lower than that in cells transfected with Kv7.2 cDNA (1.8 μg). Similarly, co-expression of Kv7.2 R325G with Kv7.3 subunits also reduced current density; more pronounced inhibitory effects were observed when Kv7.3 subunits carrying the A315T pore mutation (Kv7.3*)^18^,^19^ were used to enhance macroscopic current size. Altogether, these results suggested that Kv7.2 R325G subunits exerted significant dominant-negative effects when expressed together with Kv7.2, Kv7.3 or Kv7.3* subunits.

**Figure 2:**
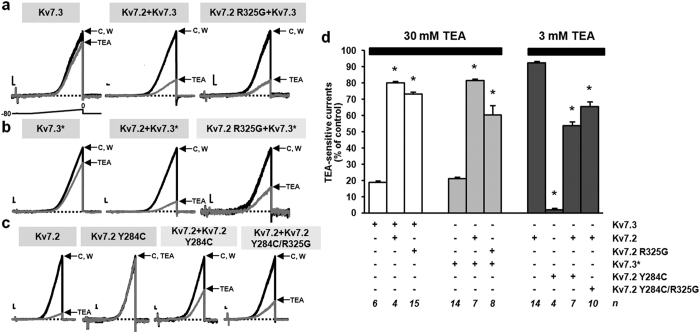
TEA-sensitivity of heteromeric channels carrying Kv7.2 R325G subunits. (**a**,**b**,**c**) Representative current responses to voltage ramps from −80 mV to 0 mV from the indicated channels recorded in control solution C, upon perfusion for 2 min with 30 mM (**a**,**b**) or 3 mM (**c**) TEA, and upon drug washout (W). Current scale: 50 pA; time scale: 200 ms. (**d**) Average data from experiments such as those shown in panels a (white bars), b (light grey bars), or c (dark grey bars). Asterisks indicate values significantly different from respective controls (leftmost bar in each group; p < 0.05). In the last row, the number of experiments performed (n), each from a separate cell, is indicated.

Pharmacological experiments with tetraethylammonium (TEA) provided further evidence for Kv7.2 R325G subunits incorporation into heteromeric channels with Kv7.2, Kv7.3 or Kv7.3* subunits. In fact, when TEA-insensitive Kv7.3 subunits^20^ were expressed together with Kv7.2 R325G subunits, currents showed a higher sensitivity to blockade by 30 mM TEA, identical to that of Kv7.2 + Kv7.3 heteromers (Fig. 2a). Similar results were observed when Kv7.3* subunits were used for these experiments (Fig. 2b). Conversely, to study heteromerization between Kv7.2 and Kv7.2 R325G subunits, a pore mutation (Y284C) was introduced to reduce TEA sensitivity^21^; heteromeric channels assembled upon Kv7.2 and Kv7.2 Y284C subunits or Kv7.2 and Kv7.2 Y284C/R325G double mutant subunits co-expression both showed an intermediate sensitivity between TEA-sensitive Kv7.2 and TEA-insensitive Kv7.2 Y284C homomeric channels (Fig. 2c). In Fig. 2d, a quantification of the pooled data from these experiments is reported. Overall, these results suggest that Kv7.2 R325G subunits were effectively incorporated into heteromeric channels with Kv7.2 or Kv7.3 subunits.

**Figure 3:**
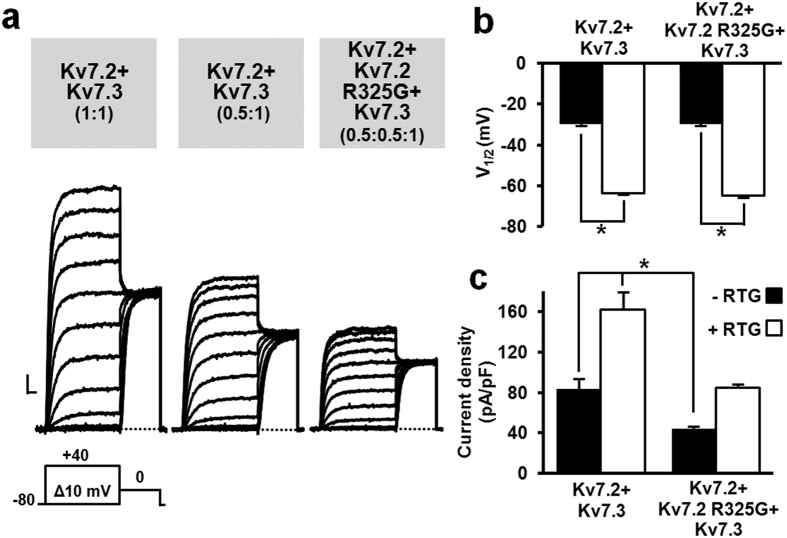
Functional characterization of heteromeric Kv7.2 + Kv7.3 channels incorporating Kv7.2 R325G subunits. (**a**) Macroscopic currents recorded in response to the indicated voltage protocol (cDNA transfection ratios are in parenthesis). Current scale: 500 pA; time scale: 200 ms. (**b,c**) Quantification of retigabine (RTG)-induced effects on activation V_½_ (**b**) and current density at 0 mV (**c**). Asterisks indicate values significantly different (p < 0.05) from respective controls. For the data shown in panels b and c, the number of experiments (n) is 14 and 20 in the absence (filled bars) and in the presence of RTG (empty bars), respectively.

To reproduce the genetic balance of Kv7.2-EE-affected patients who carry a single mutant Kv7.2 allele, and considering that most of I_KM_ is underlined by the heteromeric assembly of Kv7.2 and Kv7.3 subunits^5^, patch-clamp recordings were also performed in CHO cells co-transfected with Kv7.2 R325G together with Kv7.2 and Kv7.3 cDNAs at a 0.5:0.5:1 ratio. Macroscopic current density from Kv7.2 + Kv7.2 R325G + Kv7.3-transfected cells was smaller than that recorded from cells transfected with Kv7.2 + Kv7.3 cDNAs, both at 1:1 and 0.5:1 ratios (Fig. 3a), a result again consistent with a dominant-negative effect exerted by mutant subunits on heteromeric channel function. Current densities (in pA/pF) were 124.5 ± 4.4 (n = 25), 83.2 ± 4.1 (n = 33), and 55.5 ± 6.1 (n = 33) in Kv7.2 + Kv7.3- (1:1 ratio), Kv7.2 + Kv7.3- (0.5:1 ratio), and Kv7.2 + Kv7.2 R325G + Kv7.3- (0.5:0.5:1 ratio) expressing cells, respectively (p < 0.05 among each other). In both Kv7.2 + Kv7.3- and Kv7.2 + Kv7.2 R325G + Kv7.3-expressing cells, the Kv7 activator retigabine^22^ (RTG, 10 μM) shifted by a similar extent in the hyperpolarizing direction the voltage-dependence of channel activation (Fig. 3b), and potentiated maximal current density (Fig. 3c); as a result, current density from of Kv7.2 + Kv7.2 R325G + Kv7.3 heteromeric channels during retigabine exposure was identical to that of Kv7.2 + Kv7.3-expressing cells (Fig. 3c).

### The Kv7.2 R325G mutation affects PIP_2_-dependent regulation

**Figure 4:**
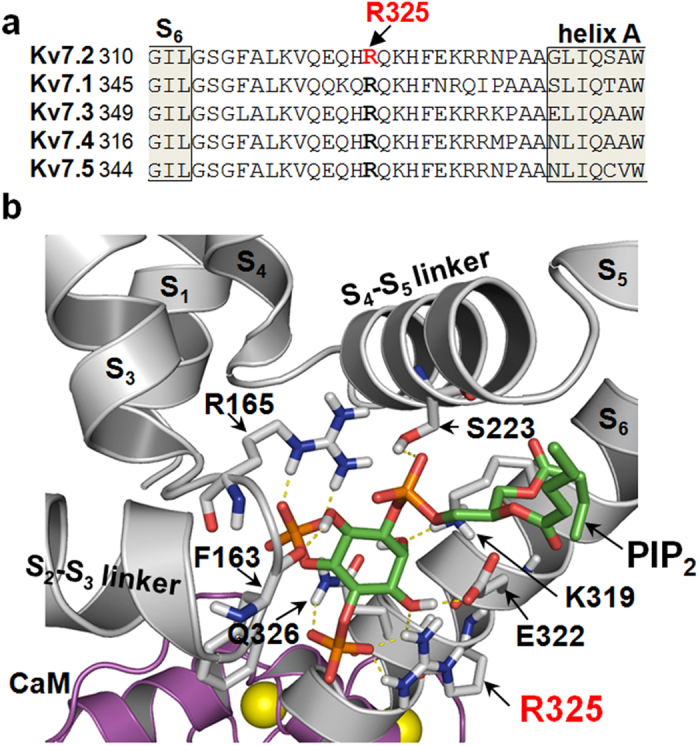
Location of the R325 residue within a PIP_2_-binding pocket in a Kv7.2 subunit. (**a**) Partial primary sequence alignment (from the end of S_6_ to the beginning of the A helix) among Kv7 subunits. (**b**) A single PIP_2_ molecule docked onto a Kv7.2 subunit. Kv7.2 α-carbon traces are in grey; the PIP_2_ molecule is shown as sticks, and colored according to atom type (carbon, green; phosphorous, orange; oxygen, red). The CaM molecule is in purple; Ca^2+^ ions are in yellow. Dashed yellow lines indicate Kv7.2/PIP_2_ interactions occurring at distances <3 Å.

The R325 residue, highly conserved among Kv7 subunits, is located in the short linker connecting the S_6_ segment with the calmodulin (CaM)-binding A-helix of Kv7.2 C-terminus (Fig. 4a)^23^. In Kv7.1 channels, PIP_2_ binding to this proximal cytosolic linker is a critical determinant of open pore stability^24^,^25^; therefore, the potential contribution of the R325 residue to PIP_2_ binding was investigated by molecular modeling studies. In particular, we grafted a short-chain derivative of PIP_2_ (dioctanoyl-PIP_2_; diC8-PIP_2_), whose crystal coordinates were taken from the diC8-PIP_2_-bound configuration of the Kir 2.2 channel^26^ onto a Kv7.2 homology model built on a Kv7.1 homology model integrating the crystal structure of the Kv7.1 proximal C-terminus including the A and B helices^27^. Starting from this model, docking experiments were performed to find the best-scoring Kv7.2/PIP_2_ configuration. The data obtained revealed that, within this region, the negatively-charged PIP_2_ molecule is involved in an intricate network of electrostatic interactions with the side chains of residues in the S_2_-S_3_ linker (F163, R165), in the S_4_-S_5_ linker (S223), and in the pre-helix A region (K319, E322, R325, and Q326) (Fig. 4b). In particular, the R325 side chain interacts with both the 3′OH and the 4′PO_4_^2−^ of the PIP_2_ molecule; the stability of the interaction between the ζ-carbon of the Kv7.2 R325 residue and the phosphorus atom at C4′ of PIP_2_ was confirmed by molecular dynamics experiments over a 10 ns time range (Suppl. Fig. 1).

**Figure 5:**
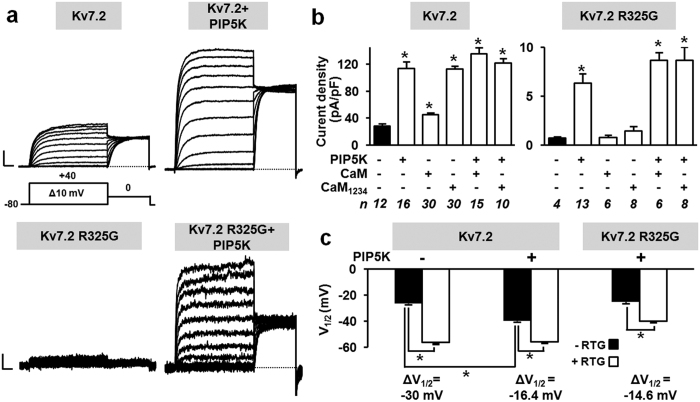
Effect of PIP5K, CaM, and CaM_1234_ on homomeric Kv7.2 and Kv7.2 R325G channels. (**a**) Macroscopic currents recorded in response to the voltage protocol shown, in the absence (left) or presence (right) of PIP5K. Current scale: 500 pA (upper panels) or 50 pA (lower panels); time scale: 200 ms. (**b**) Current densities (0 mV) from cells expressing Kv7.2 or Kv7.2 R325G channels alone or in combination with PIP5K, CaM or CaM_1234_, as indicated. In the last row, the number of experiments (n), each from a separate cell, is indicated. (**c**) Effect of retigabine (RTG, 10 μM) on activation gating (V_½_) for the indicated channels. In all panels, asterisks indicate values significantly different (p < 0.05) from respective controls.

Based on these structural hints, we next investigated whether the loss-of-function of homomeric Kv7.2 R325G channels was due to a reduced sensitivity to PIP_2_-dependent regulation; to this aim, endogenous PIP_2_ levels were increased by co-expression of type 1γ PI(4)P5-kinase (PIP5K), a PIP_2_-synthesizing enzyme^6^,^28^,^29^,^30^. In Kv7.2 channels, PIP5K enhanced current density (Fig. 5a and b), and increased current voltage-sensitivity (Fig. 5c), as described^28^,^31^. Notably, co-expression with PIP5K led to the appearance of measurable currents in cells transfected with Kv7.2 R325G cDNA (Fig. 5a), although their size was smaller than that from Kv7.2-expressing cells (Fig. 5b). No current was instead detected when only PIP5K was expressed in CHO cells (current density was 0.7 ± 0.1 pA/pF; n = 6). Currents from PIP5K-treated Kv7.2 R325G channels were blocked by 0.3 mM TEA to a similar extent as those from Kv7.2 − or Kv7.2 + PIP5K-expressing cells; the % of current blockade was 58.3 ± 1.3 (n = 8), 57.8 ± 2.6 (n = 5), or 57.0 ± 1.0 (n = 5) in cells expressing Kv7.2, Kv7.2 + PIP5K, or Kv7.2 R325G + PIP5K channels, respectively (p > 0.05). PIP5K-recovered Kv7.2 R325G currents were also sensitive to 10 μM retigabine, showing a significant leftward shift in activation gating upon drug exposure (Fig. 5c). Notably, the voltage shift in V_½_ caused by retigabine (expressed as ΔV_½_ = V_½ RTG_ − V_½ CONTROL_) in Kv7.2 R325G + PIP5K currents (−14.6 mV) was similar to that measured in Kv7.2 + PIP5K channels (−16.4 mV), both values being significantly smaller than that measured in Kv7.2 currents in the absence of PIP5K (−30.0 mV; p < 0.05) (Fig. 5c).

Co-expression with CaM or with a variant CaM unable to bind Ca^2+^  (CaM_1234_)^32^ has been shown to rescue Kv7.2 channels rendered non-functional by mutations in the CaM binding domains^33^ and to enhance Kv7.2 current density^34^ (Fig. 5b). Transfection with CaM or CaM_1234_ failed to recover currents from homomeric Kv7.2 R325G channels (Fig. 5b). Notably, CaM or CaM_1234_ were unable to further potentiate PIP5K-enhanced Kv7.2 or Kv7.2 R325G currents (Fig. 5b).

**Figure 6:**
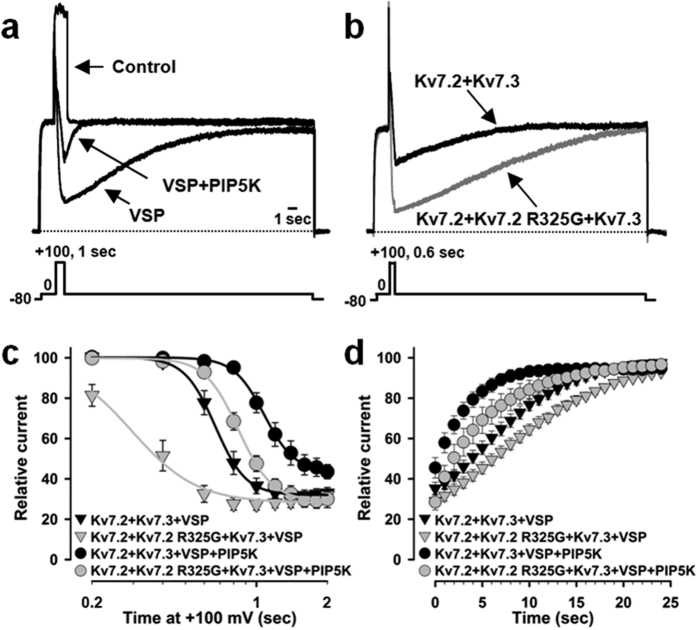
Effect of VSP on Kv7.2 + Kv7.3 and Kv7.2 + Kv7.2 R325G + Kv7.3 currents. (**a**) Currents recorded in response to the indicated voltage protocol in cells expressing Kv7.2 + Kv7.3 (control), Kv7.2 + Kv7.3 + VSP, or Kv7.2 + Kv7.3 + VSP + PIP5K, as indicated. (**b**) Currents recorded in response to the indicated voltage protocol in cells expressing VSP and Kv7.2 + Kv7.3 (black trace) or Kv7.2 + Kv7.2 R325G + Kv7.3 (gray trace) channels. (**c,d**) Time-dependence of current decrease (**c**) and recovery (**d**) in cells co-expressing the indicated channels and VSP, in the absence or in the presence of PIP5K. VSP-dependent current inhibition (**c**) was expressed as the ratio between the current values recorded at 0 mV immediately after and before the +100 mV step. Recovery (**d**) was expressed as the ratio between currents measured every second at the end and before the +100 mV depolarizing pulse. For the data shown in panels c and d, the number of experiments (n) is 11 for Kv7.2 + Kv7.3 + VSP, 11 for Kv7.2 + Kv7.3 + VSP + PIP5K, 12 for Kv7.2 + Kv7.2 R325G + Kv7.3 + VSP, and 12 for Kv7.2 + Kv7.2 R325G + Kv7.3 + VSP + PIP5K.

To deplete membrane PIP_2_ levels, a voltage-sensitive phosphatase (VSP) from zebrafish^35^ was used; this phosphatase is activated by strong depolarizations (≥100 mV), leading to a reduction of the plasma membrane content of PIP_2_ and to an inhibition of Kv7 channels^4^,^35^,^36^; membrane repolarization turns off the phosphatase, leading to PIP_2_ re-synthesis and Kv7 current recovery. In Kv7.2 + Kv7.3 channels, depolarizing pulses to +100 mV of increasing length (0.6–2 sec) time-dependently suppressed currents at 0 mV (Fig. 6a and c); the time constant of current decline at +100 mV was 0.37 ± 0.04 sec (Fig. 6d), as previously reported (0.4 sec)^29^. Current suppression occurring upon VSP activation was counteracted by PIP5K co-expression (Fig. 6a and c). After VSP turnoff by membrane repolarization, Kv7.2 + Kv7.3 current recovery time constant was 10.8 ± 0.9 sec; this value was decreased (3.6 ± 0.6 s; p < 0.05) in the presence of PIP5K (Fig. 6a and d). Instead, currents from Kv7.2 + Kv7.2 R325G + Kv7.3-expressing cells were more sensitive to VSP-induced inhibition, being inhibited by +100 mV pulses lasting only 0.2 sec, a time length unable to suppress Kv7.2 + Kv7.3 currents (Fig. 6c). PIP5K co-expression reduced current sensitivity to VSP also in Kv7.2 + Kv7.2 R325G + Kv7.3-expressing cells (Fig. 6c). On the other hand, after VSP switch-off, Kv7.2 + Kv7.2 R325G + Kv7.3 currents recovered more slowly than Kv7.2 + Kv7.3 currents, both in the absence and in the presence of PIP5K; in fact, the current recovery time constants of Kv7.2 + Kv7.2 R325G + Kv7.3 channels were respectively 6.7 ± 1.2 sec and 16.9 ± 1.7 sec with or without PIP5K (p < 0.05 versus the respective value in Kv7.2 + Kv7.3 channels; Fig. 6b and d). Altogether, these data provide evidence that the R325G mutation in Kv7.2 reduces PIP_2_ sensitivity, and that an increased cellular PIP_2_ levels can rescue the loss-of-function effect prompted by this mutation.

### The R325G mutation does not interfere with the subcellular localization of Kv7.2 subunits in rat hippocampal neurons *in vitro*

**Figure 7:**
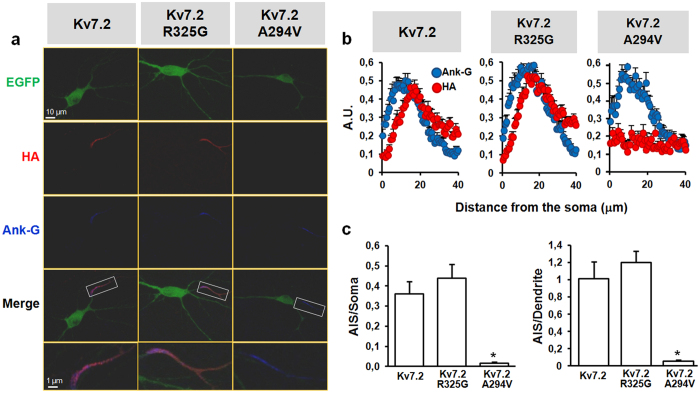
AIS localization of Kv7.2 R325G subunits. (**a**) Representative images of primary rat hippocampal neurons transfected with Kv7.3 and the EGFP-Kv7.2-HA-, EGFP-Kv7.2 R325G-HA-, or EGFP-Kv7.2 A294V-HA-expressing plasmids revealed by anti-HA (red; before permeabilization) or anti-ank-G (blue; after permeabilization) antibodies. In green is the EGFP fluorescence. Lower panels are magnifications of the boxed regions. (**b**) Quantification of the intensity (expressed as arbitrary units, A.U.) of the HA (red) and Ank-G (blue) fluorescence signals for Kv7.2 (n = 20), Kv7.2 R325G (n = 18), and Kv7.2 A294V (n = 15) subunits, measured on a 40 μm-long axonal region starting from the soma, as described in *Methods*. (**c**) Quantification of AIS/Soma and AIS/Dendrite fluorescence ratios for Kv7.2, Kv7.2 R325G, and Kv7.2 A294V subunits, calculated as described in *Methods*. Asterisks indicate values significantly different (p < 0.05) from respective controls.

In neurons, Kv7.2 and Kv7.3 subunits are primarily expressed at the axon initial segment (AIS)^37^,^38^. Epilepsy-causing mutations in Kv7.2 may interfere with such AIS targeting^39^,^40^. To assess whether the Kv7.2 R325G mutation also prompted similar effects, wild-type or mutant Kv7.2 subunits were expressed (together with Kv7.3 subunits) by transient transfection in embryonic rat hippocampal neurons. Confocal immunofluorescence experiments in non-permeabilized neurons revealed that plasma membrane expression of Kv7.2 and Kv7.2 R325G subunits could be almost exclusively detected at the Ankyrin-G-positive AIS (Fig. 7a and b); both wild-type and mutant Kv7 subunits displayed an identical AIS expression pattern (Fig. 7c), with immunoreactivity steeply increasing within the more distal regions of the AIS (Fig. 7b), as reported for native channels in rat neocortical neurons^38^. By contrast, Kv7.2 subunits carrying a different EE-associated variant affecting a pore residue (A294V) failed to localize at the AIS, as previously described^39^ (Fig. 7).

## Discussion

### The primary defect of Kv7.2 R325G channels is a decreased PIP_2_ sensitivity

Currents carried by all five members of the Kv7 family are characterized by their absolute functional requirement for PIP_2_^3^,^41^,^42^. In the present experiments, homomeric channels formed by Kv7.2 subunits carrying a mutation (R325G) found independently in four individuals affected with Kv7.2-EE^15^,^16^,^17^, were non functional, despite being expressed at the plasma membrane; Kv7.2 R325G subunits strongly suppressed channel function when incorporated into heteromers with Kv7.2, Kv7.3 or Kv7.3* subunits. The lack of Kv7.2 R325G homomeric channel function was partially rescued by co-expression with PIP5K, a lipid kinase which elevates PIP_2_ concentration in the plasma membrane to millimolar levels^28^,^30^ and reduces the ability of G_q_-coupled receptors to suppress Kv7 currents^36^, strongly suggesting that the primary dysfunction triggered by the R325G mutation is a drastic reduction in Kv7.2 sensitivity to endogenous levels of PIP_2_. Consistent with this view is also the decreased potency shown by the water-soluble PIP_2_ analogue diC8-PIP_2_ in activating Kv7.2 channel carrying the R325A mutation^43^. Noteworthy, at variance with Kv7.2 R325G channels, homomeric Kv7.2 R325A channels, similarly to Kv7.1 channels carrying the equivalent R360A mutation both in the absence^44^ or in the presence^45^ of KCNE1 subunits, were functional, although they displayed a reduced current amplitude^43^,^46^; the α-helix-stabilizing propensity of alanine relative to glycine^47^ provides a plausible explanation for such relevant functional difference. Noteworthy, all Kv7.2 missense variants causing EE (including the c.973A > G leading to the R325G mutation herein investigated) are de novo substitutions of a single nucleotide^7^,^48^. Instead, a substitution of at least two nucleotides would be needed to generate the arginine-to-alanine mutation studied by Telezhkin^43^; thus, the R325A mutation is less likely to occur *in vivo*.

Kv7.2 R325G mutant subunits, when incorporated in heteromeric channels with Kv7.2 and Kv7.3 subunits at a ratio reproducing the genetic balance of epilepsy-affected patients, exerted dominant-negative effects, enhanced current suppression by PIP_2_ depletion with VSP, and slowed current recovery kinetics after VSP turnoff; all these results, beside confirming the molecular mechanism of the primary dysfunction, also provide strong support for a significant role of the decreased PIP_2_ sensitivity in severe epilepsy pathogenesis in patients carrying the R325G variant.

### Mechanistic and structural consequences of the reduced PIP_2_ sensitivity of Kv7.2 R325G subunits

In Kv7.1 channels, PIP_2_ is not required for voltage-sensing domain (VSD) activation, being rather indispensable for coupling VSD activation to pore opening^25^. In particular, molecular dynamics experiments revealed that PIP_2_ stabilizes the open pore configuration by reducing the electrostatic repulsion among positively-charged residues in the S_4_-S_5_ linker, in S_6_, and in the proximal C-terminus immediately past S_6_^24^. Unfortunately, a truncated version of the Kv7.1 channel (residues 122 to 358) was used in these experiments^24^; thus, the potential role of the Kv7.2 R325 residue (corresponding to R360 in Kv7.1) could not be evaluated.

In Kv7.2 channels, as herein confirmed, PIP_2_ up-regulates current density and facilitates voltage-dependent opening^6^,^49^; dynamic repositioning of PIP_2_ from the VSD to the open pore gate occurs during activation^49^, whereas the reverse movement correlates with channel deactivation^46^. Thus, a critical PIP_2_ binding site in Kv7.1 and Kv7.2 channels is located at the VSD-pore domain interface, where PIP_2_ headgroups are engaged in electrostatic interactions with basic residues in the VSD and the proximal C-terminus (including the S_6_ gate)^3^,^41^, thereby bridging these two domains and providing structural stabilization^1^. The present modeling studies confirm the critical contribution of the R325 residue to PIP_2_ binding in the proximal C-terminal pocket in Kv7.2 subunits; substitution of the positively-charged arginine with a smaller, non-polar glycine may significantly weaken such structural stabilization, impeding the translation of the electromechanical forces triggered by VSD displacement into pore opening. Direct crystallographic evidence for a contribution to PIP_2_ binding of positively-charged residues located at the interface between the transmembrane and the cytoplasmic domains has been achieved in Kir2.2^26^, a PIP_2_-gated voltage-independent channel^50^. Finally, PIP_2_ binding to a similar region has been shown to stabilize the voltage sensor of Kv1 channels in a state of decreased voltage sensitivity, thus promoting functional changes opposite to those described for Kv7 channels^51^.

### Coordinated regulation of Kv7.2 channels by PIP_2_ and calmodulin

The ubiquitous Ca^2+^-binding protein CaM exerts a critical control over Kv7.2 channel function^23^,^52^. Changes in CaM binding and functional regulation have been described for several disease-causing Kv7.2 mutations^34^, and co-expression with CaM has been shown to rescue Kv7.2 channels rendered non-functional by mutations in helices A or B of the CaM-binding domain^33^. However, CHO cells transfection with either CaM or with a mutant CaM unable to bind Ca^2+^ (CaM_1234_)^32^ failed to recover functional homomeric Kv7.2 R325G channels; moreover, CaM or CaM_1234_ did not further potentiate PIP5K-induced current enhancement in both Kv7.2 and Kv7.2 R325G channels. These results are consistent with the hypothesis that CaM-induced enhancement of Kv7.2 macroscopic current density is mostly mediated by changes in PIP_2_ affinity^34^,^53^,^54^,^55^. CaM also regulates polarized axonal surface expression of Kv7.2 subunits^56^,^57^; normal AIS expression of heteromeric channels carrying Kv7.2 R325G subunits was observed in cultured hippocampal neurons, suggesting that no significant mutation-induced changes in CaM binding occurs, and that changes in PIP_2_-dependent regulation do not impede AIS trafficking of mutant Kv7.2 subunits.

### Pharmacological implications and conclusions

Retigabine is the prototype anticonvulsant acting as an activator of neuronal Kv7 channels (Kv7.2-5); retigabine causes a variable degree of hyperpolarization shift of the voltage dependence of channel activation, together with an increase in channel maximal opening probability^22^,^58^. The present observation that increasing cellular PIP_2_ levels with PIP5K negatively shifted the V_½_ of Kv7.2 channels (as previously described for Kv7.1^59^, Kv7.2^49^, and other heteromeric Kv7 channels^31^) and decreased retigabine-induced responses in both in Kv7.2 and Kv7.2 R325G channels, indicates that retigabine and PIP_2_ act via at least partially overlapping mechanisms to stabilize voltage-dependent pore opening; consistent with this view is the fact that PIP_2_-depleted Kv7.3 channels are insensitive to retigabine^60^.

In conclusion, the present results add severe forms of Kv7.2-related epilepsy to the growing list of channelopathies caused by changes in PIP_2_-dependent regulation^1^,^59^; the recent observation that Kv7 channels are critical determinants of the cortical excitability changes occurring upon dynamic regulation of PIP_2_ levels^31^, lends further support to the pathogenetic role of the proposed mechanism in individuals carrying the Kv7.2 R325G variant. Future studies will further explore the structural implications of the functional results herein presented, and define whether such specific molecular defect is associated with distinct clinical features.

## Methods

### Mutagenesis and heterologous expression of channel subunits

Mutations were engineered by Quick-change mutagenesis (Agilent Technologies) in a pcDNA3.1-Kv7.2 plasmid (for electrophysiological and western-blot experiments), or in a dual-tagged Enhanced Green Fluorescent Protein-Kv7.2-hemagglutinin (EGFP-Kv7.2-HA) plasmid (for immunocytochemistry experiments), as described^21^,^34^,^61^. In the EGFP-Kv7.2-HA chimeric constructs, in addition to an EGFP at the cytoplasmic N-terminus, an HA epitope was inserted in the extracellular loop that connects transmembrane domains S_1_ and S_2_ of Kv7.2 subunits, as previously described^61^,^62^. Protocols for wild-type and mutant cDNAs expression by transient transfection, as well as methods for CHO cell growth, have been previously described^34^. Total cDNA in the transfection mixture was kept constant at 4 μg, except for Western-blotting experiments (6 μg).

### Cell surface biotinylation and Western-blot

Total or plasma membrane expression of Kv7.2 or Kv7.2 R325G subunits in CHO cells was investigated by surface biotinylation and western-blotting analysis, as described^63^. Channel subunits were identified using mouse monoclonal anti-Kv7.2 primary antibodies (clone N26A/23, dilution 1:1000; Antibodies Inc., Davis, CA), followed by horseradish peroxidase (HRP)-conjugated anti-mouse secondary antibodies (clone NA931V; dilution 1:5,000; GE Healthcare, Little Chalfont, UK). Anti-Kv7.2 monoclonal antibodies were generated using a fusion protein corresponding to amino acids 1–59 of the cytoplasmic N-terminus of human Kv7.2 subunits (accession number O43526).

### Patch-clamp recordings

Macroscopic current recordings from transiently-transfected CHO cells, as well as data processing and analysis, were performed as reported^11^. In the experiments with TEA or retigabine (obtained from Valeant Pharmaceuticals, Aliso Viejo, CA), currents were activated by 3-s voltage ramps from −80 mV to 0/+40 mV at 0.1-Hz frequency. TEA blockade was expressed as the percentage of peak current inhibition produced by a 2-min drug application.

### Molecular modeling

#### Homology modeling

A homology model of a Kv7.2 subunit was generated starting from a Kv7.1 homology model which integrates the crystal structure of the Kv7.1 proximal C-terminus^27^, using the SWISS-MODEL software^64^. This model was then aligned with the crystal structure of Kir2.2 channels bound to diC8-PIP_2_^26^, using the Chimera matchmaker tool (https://www.cgl.ucsf.edu/chimera/docs/ContributedSoftware/matchmaker/matchmaker.html); the PIP_2_ crystal coordinates were then superimposed onto the Kv7.2 model. *Protein preparation.* To obtain a satisfactory starting structure for following studies, the Kv7.2 protein was prepared using the Schrodinger Protein Preparation Wizard^65^. The orientation of hydroxyl groups on S, T and Y, the side chains of N and Q residues, and the protonation state of H residues were optimized. N- and C- terminal residues were capped with acetyl and N-methyl-amide residues, respectively. The ionization and tautomeric states of H, D, E, R and K residues were adjusted to match a pH of 7.4. The structure was finally submitted to a restrained minimization (OPLS2005 force field)^66^ that was stopped when the root-mean-square deviation (RMSD) of heavy atoms reached 0.30 Å. *Docking studies.* The Schrodinger Induced Fit Docking Extended Sampling protocol^67^,^68^ was used for docking studies of PIP_2_ on the optimized Kv7.2 configuration. The docking space, centered on the PIP_2_ molecule, was defined as a 32 Å^3^ cubic box, while the diameter midpoint of docked ligands was restrained within a smaller, nested 22 Å^3^ cubic box. Residues within 10 Å of ligand poses were refined by the Prime Software (https://www.schrodinger.com/prime). *Molecular Dynamics simulations.* The stability of the best scoring PIP_2_/Kv7.2 complex was further investigated by Molecular Dynamic (MD) simulations using Desmond MD system (https://www.schrodinger.com/desmond). The simulated environment was built using the system builder utility, with the structures being neutralized by Na^+^ and Cl^−^ ions, which were added to a final concentration of 0.15 M. Simulations were run in explicit solvent, using the TIP4P water model^69^ in a Periodic Boundary Conditions orthorhombic box. A series of minimizations and short MD simulations were carried out to relax the model system, by means of a relaxation protocol consisting of six stages: (i) minimization with the solute restrained; (ii) minimization without restraints; (iii) simulation (12 ps) in the NVT ensemble using a Berendsen thermostat (10 °K) with non-hydrogen solute atoms restrained; (iv) simulation (12 ps) in the NPT ensemble using a Berendsen thermostat (10 °K) and a Berendsen barostat (1 atm) with non-hydrogen solute atoms restrained; (v) simulation (24 ps) in the NPT ensemble using a Berendsen thermostat (300 °K) and a Berendsen barostat (1 atm) with non-hydrogen solute atoms restrained; (vi) unrestrained simulation (24 ps) in the NPT ensemble using a Berendsen thermostat (300 °K) and a Berendsen barostat (1 atm). At this point, a 10 ns long MD simulation was carried out at a temperature of 300 °K in the NPT ensemble using a Nose-Hoover chain thermostat and a Martyna-Tobias-Klein barostat (1.01325 bar). Backbone atoms were constrained during the simulation (10 kcal/mol). Trajectory analyses were performed using the Desmond simulation event analysis tool.

### Neuronal cell transfection and immunocytochemistry

Hippocampal cultures were prepared from 18-day embryonic rats, as described^70^. At 8 days *in vitro* (8 DIV), neurons were co-transfected with EGFP-Kv7.2-HA (wild-type or mutant) and Kv7.3 cDNAs (ratio 1:1, total 2 μg) using Lipofectamine 2000 and immunostaining was performed at room temperature 72 hr after transfection. For surface immunostaining, neurons were fixed in 4% paraformaldehyde/4% sucrose for 10′ at 37 °C, blocked with 10% normal goat serum in PBS and incubated with rabbit anti-HA antibodies (1:60; 745500; Invitrogen) diluted in 10% normal goat serum in PBS for 1 hr. For permeabilized immunostaining, neurons were incubated with mouse monoclonal anti-ankyrin-G antibodies (1:200; clone 106/36; Millipore) diluted in permeabilizing buffer (15 mM phosphate buffer pH 7.4 containing 0.1% gelatin, 0.3% Triton X-100 and 0.4 M NaCl) for 2 hr. After PBS wash, neurons were incubated simultaneously with rabbit AlexaFluor555- and mouse AlexaFluor649-conjugated secondary antibodies (1:400 and 1:300, respectively; LifeTechnologies) for 1 hr in permeabilizing buffer. Coverslips were then mounted with moviol. Confocal image acquisition was performed on a Zeiss LSM510 Meta laser scanning microscope equipped with a 63x oil immersion lens. The ImageJ software was used for image analysis. Axonal Ank-G and HA signals were measured every 0.14 μm along a 40 μm-long region starting from the soma; values (expressed as fluorescence arbitrary units of intensity) in each neuron were normalized, and averaged every 5 points to decrease signal noise. AIS/Soma and AIS/Dendrites ratios were calculated by expressing the HA fluorescence (measured in a 20–30 μm AnkG-positive area) versus the EGFP fluorescence of a 50 μm^2^ rectangle in the soma (AIS/Soma) or versus a 25 μm-long region of the main dendrite (AIS/Dendrite)^39^.

### Animals

Experimental procedures were performed in accordance with the European Communities Council Directive (86/809/EEC) on the care and use of animals and the UK Animals (Scientific Procedures) Act 1986, and were approved by the Animal Care and Use Committee of the CNR Institute of Neuroscience.

### Statistics

Data are expressed as mean ± SEM. Each data point shown in figures or in the text is the Mean ± SEM of at least 4 determinations, each performed in a single cell or in a separate experiment. Statistically significant differences were evaluated with the Student’s t-test or with the ANOVA followed by the Student-Newman-Keuls test, with the threshold set at p < 0.05.

## Additional Information

**How to cite this article**: Soldovieri, M. V. *et al*. Early-onset epileptic encephalopathy caused by a reduced sensitivity of Kv7.2 potassium channels to phosphatidylinositol 4,5-bisphosphate. *Sci. Rep.* **6**, 38167; doi: 10.1038/srep38167 (2016).

**Publisher's note:** Springer Nature remains neutral with regard to jurisdictional claims in published maps and institutional affiliations.

## Supplementary Material

Supplementary Datasets 1 and 2
