# Supplementary material for: Early-onset epileptic encephalopathy caused by a reduced sensitivity of Kv7.2 potassium channels to phosphatidylinositol 4,5-bisphosphate
Source: Sci Rep. 2016 Dec 1;6:38167. doi: 10.1038/srep38167 (PMC5131271; doi:10.1038/srep38167)
Supplement: Supplementary Datasets 1 and 2 [file srep38167-s1.doc]

**SUPPLEMENTARY DATA SET**

**Early-onset epileptic encephalopathy caused by a reduced sensitivity of Kv7.2 potassium channels to phosphatidylinositol 4,5-bisphosphate**

Maria Virginia Soldovieri^1^*, Paolo Ambrosino^1^*, Ilaria Mosca^1^, Michela De Maria^1^,

Edoardo Moretto^2^, Francesco Miceli^3^, Alessandro Alaimo^4,5^, Nunzio Iraci^6^, Laura Manocchio^1^,

Alessandro Medoro^1^, Maria Passafaro^2^, and Maurizio Taglialatela^1,3#^

^1^Department of Health Science, University of Molise, Campobasso, Italy;

^2^CNR Institute of Neuroscience, Department of Medical Biotechnology and Translational Medicine (BIOMETRA), University of Milan, Milan, Italy;

^3^Department of Neuroscience, University of Naples Federico II, Naples, Italy;

^4^Center for Integrative Biology (CIBIO), University of Trento, Trento, Italy;

^5^Instituto Biofisika (UPV/EHU, CSIC), Leioa, Spain;

^6^Department of Pharmacy, University of Salerno , Fisciano, Salerno, Italy

^#^Corresponding Author: Maurizio Taglialatela, MD PhD

Department of Neuroscience, University of Naples Federico II.

Via Pansini 5, 80131 – Naples, ITALY.

Tel. (+39) 081-7463316; Fax: (+39) 081-7463323

Email: mtaglial@unina.it

*MVS and PA contributed equally to this work.

Key words: Epileptic encephalopathy; Kv7.2 channels; Axon initial segment; Retigabine; PIP_2_

Conflict of Interest: The authors declare no competing financial interests


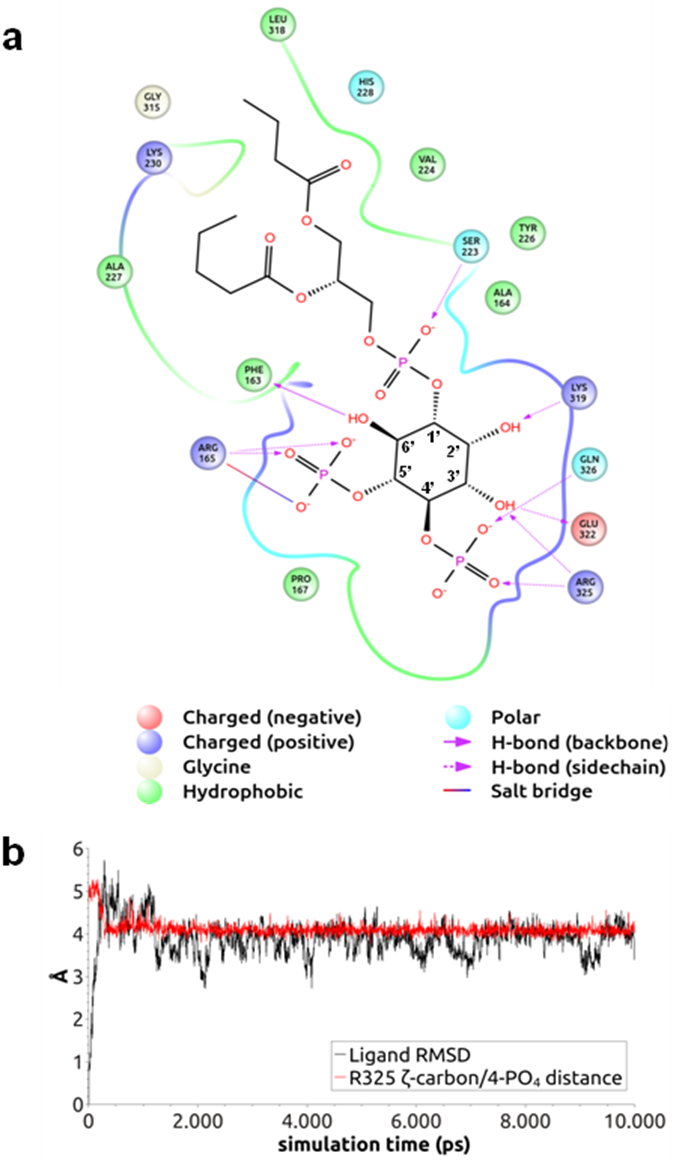


**Suppl. Fig. 1. Docking and molecular dynamics results for PIP2 interaction with a post-S6 pocket in a Kv7.2 subunit. (**a) Schematic bidimensional representation of the interactions between PIP_2_ and Kv7.2 residues predicted by the docking experiments. (**b**) Stability of the interaction between the ζ-carbon of the Kv7.2 R325 residue and the phosphorus atom at C4' of PIP2 revealed by molecular dynamics experiments over a 10 ns time range. Black line: PIP_2_RMSD as a function of simulation time. Red line: Distance between R325 ζ-carbon and phosphorus atom at C4' as a function of simulation time.


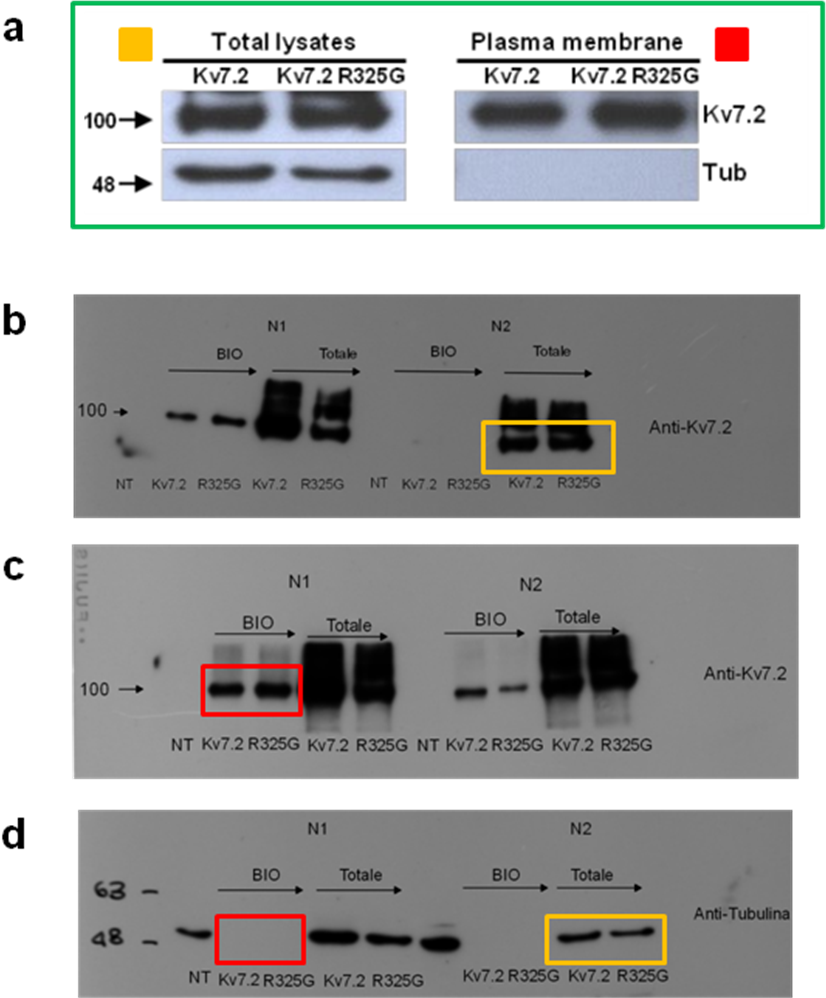


**Suppl. Fig. 2. Full-length blots of cropped images shown in Fig. 1B.** a, Fig. 1B of the manuscript, containing images cropped from the full-lenght blots reported below. Orange symbol: total lysates; red symbol: plasma membrane proteins. b and c, Full-lengh blots containing the original Kv7.2 signals in total lysates (b, orange rectangle) or in plasma membrane fractions (BIO; b, red rectangle) at different exposures. NT: non transfected cells (negative control of anti-Kv7.2 antibodies). d, Full-lengh blot containing the original tubulin signals from the same total lysates (orange rectangle) or plasma membrane fractions (BIO, red rectangle) shown in b and c, respectively.
